# Trends and risk factors associated with hepatitis A severity and mortality in French hospitals: a national population-based study, 2013 to 2024

**DOI:** 10.2807/1560-7917.ES.2026.31.39.2500858

**Published:** 2026-10-01

**Authors:** Anne-Marie Roque-Afonso, Charlotte Mouliade, Lucia Parlati, Nathalie Goutté, Julie Figoni, Samir Bouam, Philippe Sogni, Vincent Mallet, on behalf of the Demosthenes research group

**Affiliations:** 1 Université Paris Saclay, INSERM U1193; AP-HP, Hôpital Paul Brousse, National Reference Center for Hepatitis A, Villejuif, France; 2 Université Paris Cité, Paris, France; 3 AP-HP. Centre Université Paris Centre, Groupe Hospitalier Cochin Port Royal, DMU Cancérologie et spécialités médico-chirurgicales, Service des Maladies du foie, Paris, France; 4 INSERM U1193; APHP, Hôpital Paul Brousse, Direction des soins, Villejuif, France; 5 Santé Publique France, Saint-Maurice, France; 6 AP-HP. Centre Université de Paris, Groupe Hospitalier Cochin Port Royal, DMU PRIME, Service d’Information Médicale, Paris, France; 7 Members of the Demosthenes research group are acknowledged at the end of the article

**Keywords:** Hepatitis A, Hospital data, France / epidemiology, Disease Severity, Mortality, Risk Factors, Comorbidities

## Abstract

**BACKGROUND:**

As hepatitis A (HA) incidence declines in Europe and HA virus infections occur later in life, severe disease frequency may increase.

**AIM:**

Among patients hospitalised for HA in France during 2013−2024, we investigated disease severity and mortality associated-risk factors and temporal trends.

**METHODS:**

International Classification of Diseases, 10th Revision codes B150 or B159 as primary discharge diagnosis enabled identifying HA cases from the National Discharge Data Set. Risk factors for severity (hepatic and/or extrahepatic organ failure within 12 weeks post-admission) and mortality were assessed using adjusted odds ratios in original and propensity-matched samples. Trends were determined across five periods covering an HA epidemic in 2017 and the COVID-19 pandemic, with 2013–2016 as reference.

**RESULTS:**

Among 7,923 cases (60.6% male, 39.4% female; median age: 30 years), 28.7% (n = 2,277) developed severe HA, and 1.30% (n = 103) died. Severe HA was associated with older age (+ 15% per decade over 30 years, p < 0.001), male sex (+ 38%, p < 0.001), smoking (+ 23%, p = 0.031), risk factors for chronic liver disease (+ 29%, p = 0.041), and cirrhosis (+ 40%, p = 0.054). Mortality was associated with cirrhosis (2.90-fold increase, p = 0.004), Charlson Comorbidity Index (CCI) ≥ 3 (12.3-fold increase, p < 0.001), and modestly with age (+ 24%, p = 0.005), reaching + 90% (p < 0.001) in models without CCI, indicating a considerable comorbidity effect. From 2013–2016, severe HA and HA-related death risks respectively increased 57% (p < 0.001) and 1.96-fold (p = 0.016) in 2021–2024.

**CONCLUSIONS:**

Severe HA frequency and mortality increased in French hospitals since 2013. Age and male sex were associated with severe disease, and high CCI with mortality. A more extensive, or even universal, vaccination policy could be considered.

**Figure:**
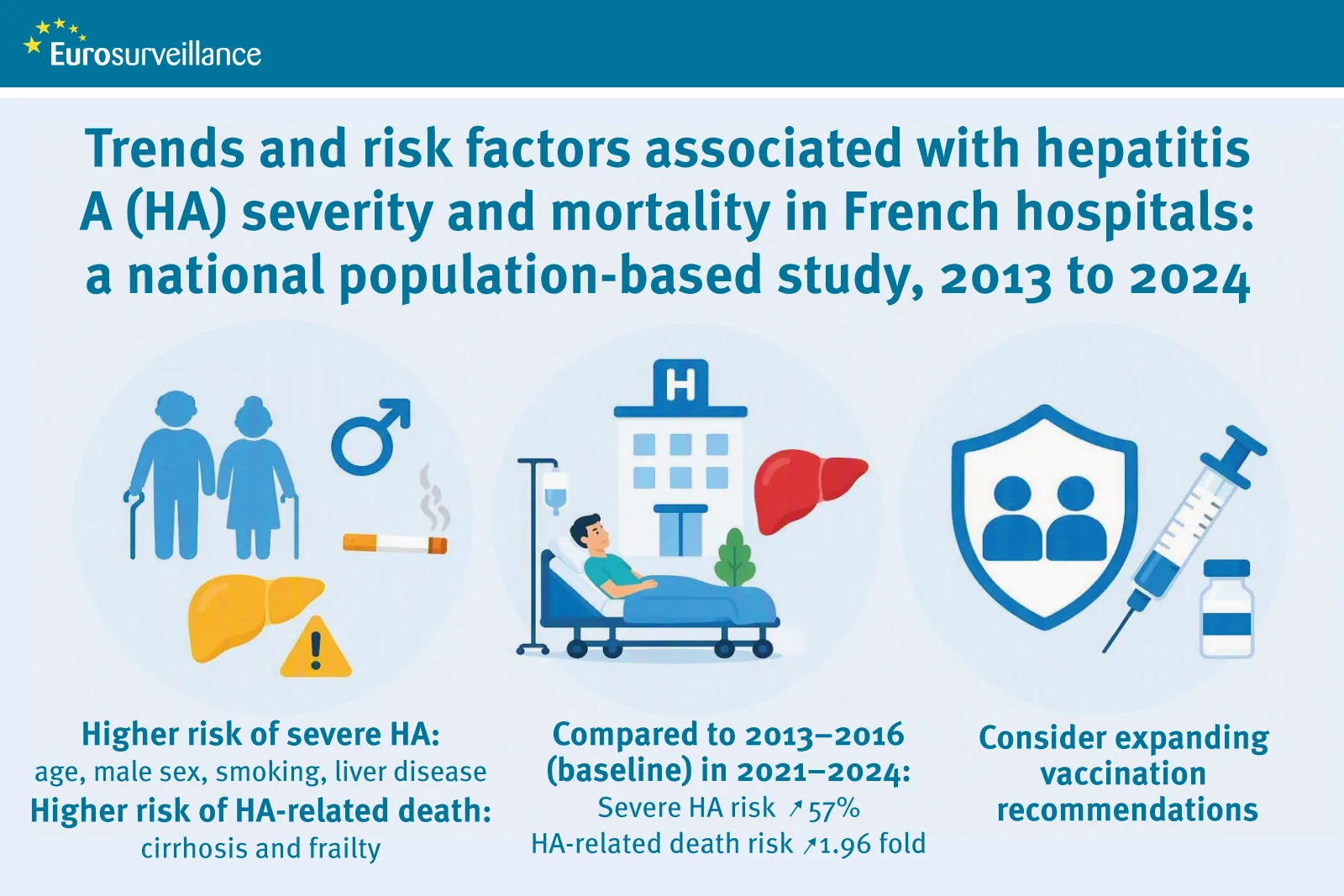

**Key public health message**

**What did you want to address in this study and why?**

As hepatitis A incidence declines in Europe and infections with hepatitis A virus occur later in life, more people experience severe disease, particularly during outbreaks involving adults. We wanted to analyse risk factors associated with disease severity and mortality in patients hospitalised in France from 2013 to 2024 as well as trends of hepatitis A hospitalisations with severe disease or mortality.

**What have we learnt from this study?**

Occurrence of severity and mortality among patients with hepatitis A has risen over the past 12 years. Advanced age, male sex, risk factors for chronic liver disease, cirrhosis, and smoking increased the risk of severe disease i.e. hepatic or extrahepatic organ failure during hepatitis A virus infection. Hepatitis A-related mortality was associated with cirrhosis and a high comorbidity burden.

**What are the implications of your findings for public health?**

In France, where hepatitis A endemicity is low, the higher frequency of more severe hepatitis A presentation calls for optimising care for identified risk groups and strengthening targeted prevention strategies. Expanding vaccination recommendations to more risk categories or even a universal vaccination policy could be considered. The current study could be repeated in countries with a similar epidemiology to France to confirm its findings generalisability.

## Introduction

Hepatitis A virus (HAV) is transmitted via the faecal-oral route from person-to-person or through contaminated food or water, and therefore closely linked to a country's socioeconomic and hygiene conditions. Hepatitis A (HA) is mainly asymptomatic in children, but the frequency and severity of symptoms increase with age. On rare occasions, patients develop fulminant hepatitis, which can lead to multiple organ failure with a high mortality rate, reaching up to 5% in people over 50 years of age, particularly when liver transplantation (LT) is not an option [1]. 

Over the past three decades, HA has ranked as the leading cause of acute viral hepatitis, with a global increase in the number of cases from 139.54 million in 1990 to over 160 million in 2021 [2,3]. However, the age-standardised incidence tends to decrease, and there has been a dramatic decline in age-standardised disability-adjusted life years (DALY) rates and mortality rates, possibly reflecting advances in the management and treatment of organ failure [2,4]. In most European countries, the incidence of HA is low and declining, and a growing proportion of the population is susceptible to HAV. This situation leads to repeated outbreaks, either food-borne (in particular related to imported food), or due to human-to-human transmission among under-vaccinated risk groups, such as men who have sex with men (MSM), travellers to endemic areas, or vulnerable populations at high risk such as people who experience homelessness or people who use drugs [5,6]. In addition, as the average age of infection shifts upward, severe illness becomes more common, and hospitalisation rates can be high during outbreaks [7].

In France, HA surveillance since 2006 has shown a low incidence of disease (< 2/100,000 inhabitants) with a downward trend until a major epidemic occurred in 2017, mainly affecting MSM [8], and a new upward trend was noted from 2023 onwards (data available from [9]). Surveillance is based on mandatory reporting but is subject to under-reporting [10,11], and provides limited clinical data, with no information on severity or case-fatality. However, notifications specify if a case is in hospital or not. The objective of this study was to describe trends in the incidence of hepatitis A in French hospitals between 2013 and 2024, and to identify demographic and clinical factors associated with severity and in-hospital mortality, based on national hospital discharge data.

## Methods

### Data collection and study population

Data were sourced from the Programme de Médicalisation des Systèmes d’Information (PMSI; the French National Hospital Discharge Data Set), a dataset which has prior demonstrated high validity for hard outcomes requiring hospital care, as further clarified by the ‘Additional information’ in the Supplementary document [12]. The database contains anonymised, standardised discharge summaries providing detailed patient demographics (age, sex as binary data, postal code of residency), primary and associated discharge diagnoses coded using the International Classification of Diseases, 10th Revision (ICD-10) [13], medical procedures, discharge dates, length of stay, and entry and discharge modes (e.g. emergency admission, transfer, discharge home, or in-hospital death). During the observation period, the entire French population (68.4 million in 2022) had equal access to universal, tax-financed healthcare services. Each patient’s historical and underlying conditions were identified longitudinally by linking all hospital stays belonging to the same individual over the study period using unique anonymous identifiers [14]. 

As hospital discharge data are recorded for all hospitalisations in France, missingness was minimal for primary outcomes and key covariates, all identified using ICD-10 codes listed in Supplementary Table S1. Covariates included age, sex, alcohol use disorders (AUD), smoking habits, obesity (body mass index > 30 kg/m^2^), type-2 diabetes mellitus, cirrhosis, human immunodeficiency virus (HIV) infection, risk factors for chronic liver disease, severe comorbidities assessed using the Charlson Comorbidity Index (CCI), and socioeconomic deprivation measured by the French Deprivation Index (Fdep) [15]. The AUD were identified in the same way as in global burden studies and previous research [12,16]. Risk factors for chronic liver disease included all well identified causes of such diseases (chronic hepatitis B and C, non-viral causes of chronic liver disease, i.e. congenital malformations, genetic disorders other than cystic fibrosis, autoimmune liver diseases, and disturbances in iron or copper metabolism). The CCI, widely used to predict a patient’s 1-year survival probability by quantifying the burden of comorbid conditions, assigns weighted scores to specific chronic diseases, and the sum of these weights, calculated by using developed ICD-10 coding algorithms, gives the patient’s score [17]. A score ≥ 3 indicates moderate to high comorbidity burden and therefore greater frailty. The Fdep was used to assess spatial socioeconomic and health inequalities, with scores divided into quintiles [15]: a score ≥ Q4 indicates greater socioeconomic deprivation.

We performed complete case analyses for multivariable models, and no data imputation was performed. We identified all patients recorded with HA, i.e. with ICD-10 codes B150 or B159 as a primary or associated discharge diagnosis, between January 2013 and December 2024 (n = 13,480). To increase specificity, we included only patients with HA as primary discharge code (i.e. n = 7,923; 58.8%), as described in the Supplementary Figure S1 flowchart.

Data from national surveillance through mandatory reporting [9] were used to confirm the trends identified by our analysis of hospital data, namely variations in incidence, and variations in case severity, as reflected within the notification data by the hospitalisation rate among reported cases, obtained by dividing the number of reported cases notified as being in hospital by the total number of reported cases, as a percentage.

### Outcome measures

Our primary objective was to measure the incidence of severe HA and HA-related death over time. Severe HA was defined as hepatic and/or extrahepatic organ failure within 12 weeks of admission with the first record of HA as primary discharge diagnosis, and death was not included in the definition of severity. The codes used to define severe HA are highlighted in yellow in Supplementary Table S1. These codes correspond to diagnosis codes of organ failure and to organ support therapeutic procedures, including LT. Definition of HA-related mortality was death within 12 weeks of admission for HA.

### Data analysis

The primary explanatory variable was the period of observation. Periods were defined to take into account two major events that occurred during the 12-year study: the HAV epidemic in 2017, mainly affecting MSM [8] and the COVID-19 pandemic. The periods were (i) before the outbreak in MSM (period A: Jan 2013−Dec 2016), considered as reference period, (ii) the outbreak in MSM (period B: Jan 2017−Jun 2018), (iii) after the outbreak in MSM until the COVID-19 pandemic (period C: Jul 2018−Dec 2019), (iv) the COVID-19 pandemic, as defined for France (period D: Jan 2020−Jun 2021) [18], and (v) after the COVID-19 pandemic (period E: Jul 2021−Dec 2024). Other explanatory variables were the key covariates referred to above.

Incidence rates per 100,000 population were calculated using population size data from the Institut National de la Statistique et des Études Économiques (https://www.insee.fr/fr/statistiques/1893198). Standardised rates were computed by direct standardisation using the European Standard Population 2013 (ESP2013) [19] as the reference; the detailed methodology is provided in the Supplementary Methods. Crude incidences stratified by age group, averaged between the sexes, are also presented for descriptive purposes and should be interpreted as unadjusted estimates. The same applies to the crude incidence of HA in the general population, calculated from numerators derived from mandatory reporting.

Adjusted odds ratios (aOR) from multivariable binary logistic regression models were used to measure the strength of associations. Variables with nominal 2-tailed p values < 0.05 were included in the multivariable models. Propensity scores were estimated considering the likelihood of severe HA or HA-related death, using all studied covariates to adjust for potential confounding factors across time periods. A full matching approach was then applied, whereby all individuals were retained and grouped into matched sets containing at least one patient from each period, with variable matching ratios, to minimise differences in covariate distributions between periods while preserving sample size [20].

In a sensitivity analysis, the multivariable models for severe HA and for HA-related death, and the corresponding propensity-score full-matching models, were refitted after excluding the CCI. This was done because the CCI is age-adjusted, overlaps with individual comorbidities already entered as covariates and, because the severity endpoint incorporates organ-support procedures, the CCI may partly capture limitation of care rather than disease severity. Models are reported with and without the CCI in Supplementary Table S4. Statistical tests were two-tailed with p < 0.05 considered as significance. Analyses were performed using R statistical software (R version 4.4.0 *Puppy Cup*) [21].

## Results

### Patients’ characteristics and hepatitis A incidence trends in French hospitals during 2013–2024

A total of 7,923 hospitalisations with HA as primary discharge code were recorded between 2013 and 2024. Median age was 30.0 years (interquartile range (IQR): 18–48 years), 60.65% were male and 39.35% were female. Risk factors for chronic liver disease were present in 12.20%, namely AUD (5.83%), cirrhosis (2.21%), and risk factors for chronic liver diseases, as defined in the methods (4.13%); 13.10% had obesity or type-2 diabetes; prevalence of HIV infection was 3.89%; 11.27% had a CCI ≥ 3 (Table 1).

**Table 1:** Characteristics of patients hospitalised for hepatitis A across different periods, France, 2013–2024 (n = 7,923 patients)

Characteristics	Overall		Period A Pre-MSM outbreak Jan 2013–Dec 2016		Period B MSM outbreak Jan 2017–Jun 2018		Period C Post-MSM outbreak Jul 2018–Dec 2019		Period D COVID-19 pandemic Jan 2020–Jun 2021		Period E Post-COVID-19 pandemic Jul 2021–Dec 2024		p value^a^
	Number	%	Number	%	Number	%	Number	%	Number	%	Number	%	
Number included in analysis	7,923	100	2,105	26.6	2,418	30.5	1,350	17.0	414	5.22	1,636	20.6	
**Age in years, median (IQR)**	30.0 (18.0–48.0)		27.0 (14.0–48.0)		34.0 (25.0–48.0)		27.0 (15.0–47.0)		38.0 (19.0–61.0)		28.0 (15.0–48.0)		< 0.001
**Male sex^b^**	4,805	60.65	1,094	51.97	1,843	76.22	740	54.81	193	46.62	935	57.15	< 0.001
**Smoking habits**	666	8.41	148	7.03	274	11.33	107	7.93	37	8.94	100	6.11	< 0.001
**Alcohol use disorders**	462	5.83	113	5.37	195	8.06	68	5.04	22	5.31	64	3.91	< 0.001
**Obesity^c^**	649	8.19	188	8.93	187	7.73	110	8.15	47	11.35	117	7.15	0.039
**Type-2 diabetes**	389	4.91	125	5.94	94	3.89	58	4.30	29	7.00	83	5.07	0.004
**Liver risk factors^d^**	327	4.13	105	4.99	84	3.47	58	4.30	20	4.83	60	3.67	0.090
**Cirrhosis**	175	2.21	60	2.85	43	1.78	27	2.00	13	3.14	32	1.96	0.075
**HIV infection**	308	3.89	31	1.47	242	10.01	21	1.56	1	0.24	13	0.79	< 0.001
**CCI ≥ 3**	893	11.27	211	10.02	188	7.78	157	11.63	105	25.36	232	14.18	< 0.001
**Fdep ≥ Q4^e^**	3,191	41.37	880	43.48	842	35.47	581	44.05	168	42.42	720	45.00	< 0.001
**Severe HA**	2,277	28.74	481	22.85	805	33.29	400	29.63	109	26.33	482	29.46	< 0.001
**HA-related death**	103	1.30	24	1.14	15	0.62	14	1.04	12	2.90	38	2.32	< 0.001

CCI: Charlson Comorbidity Index; Fdep: French deprivation index; HA: hepatitis A; HIV: human immunodeficiency virus; IQR: interquartile range; Q4: fourth quarter; MSM: men who have sex with men.

^a^ Kruskal−Wallis rank sum test for continuous variables; Pearson’s chi-squared test for categorical variables.

^b^ There were 3,118 female patients (39.35%).

^c^ Body mass index > 30 kg/m^2^.

^d^ These are risk factors for chronic liver disease, as detailed in the Methods’ section.

^e^ The denominators of the percentages were adjusted due to missing values associated with the Fdep variable. These concerned a total of 210 patients who were from overseas territories or non-French residents, including 81 in period A, 44 in period B, 31 in period C, 18 in period D and 36 in period E.

Severe HA was defined as hepatic and/or extrahepatic organ failure within 12 weeks of admission.

The annual number of hospitalised HA cases ranged from 246 in 2020 to 1,997 in 2017, as illustrated in Supplementary Figure S2, and standardised incidence ranged from 0.36 in 2020 to 3.22/100,000 in 2017 (Figure 1).

**Figure 1:**
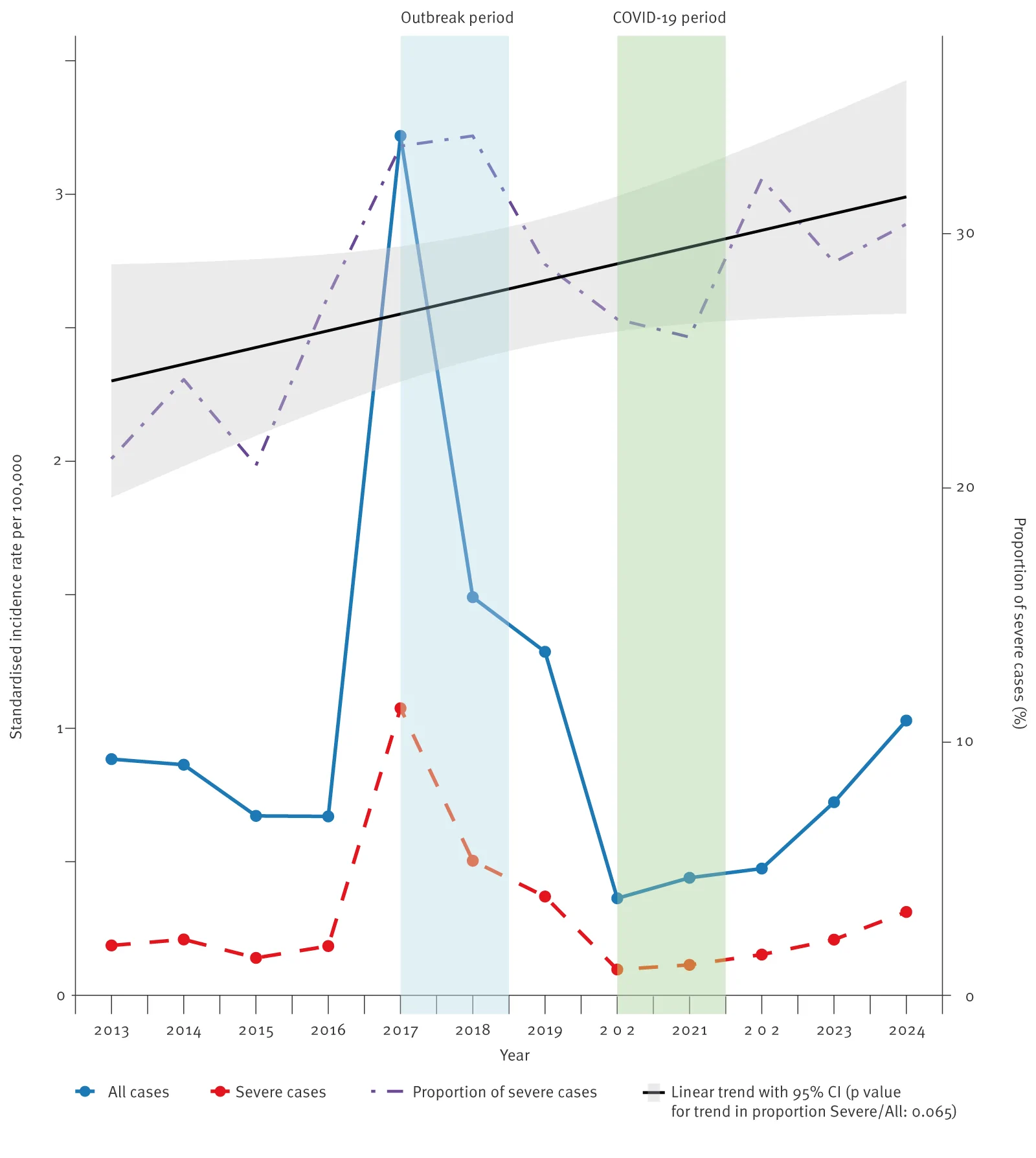
Standardised incidence rates of hepatitis A (n = 7,923 inpatients) and of severe hepatitis A cases in hospitals (n = 2,277 inpatients), per 100,000 population, France, 2013–2024

CI: confidence interval.

The data concern France, including Metropolitan France and Overseas Departments.

Standardised incidence rates for all HA cases (solid blue line) and for severe cases (dashed red line) are presented. The proportion of severe cases is on the right-hand axis (dotted purple line), with the black line and grey shading indicating the linear trend (p = 0.065) and its 95% CI, respectively. Shaded areas indicate the outbreak in MSM (blue) and the COVID-19 pandemic (green).

Incidence rates varied greatly not only from year to year but also according to sex or age group (Figure 2). While annual age-standardised incidence rates among men appeared to be higher overall than those observed among women, the trends in cases among men and women were consistent with one another over the period from 2013 to the end of 2016, during which both groups showed a slight decline. From the start of the COVID-19 pandemic, incidence rates among male and female patients also appeared to follow similar trends, reaching historically low levels in 2020 before rising again thereafter. A notable difference between the sexes nevertheless emerged between 2017 and 2019, a period during which age-standardised annual incidence rates were significantly higher among men than among women (incidence rate ratio: 2.25; 95% confidence interval (CI): 2.10–2.41; p < 0.001). A peak for both sexes at 5.2 per 100,000 for males and 1.3 per 100,000 per females occurred in 2017, coinciding with a hepatitis A outbreak among MSM (Figure 2A). With regard to crude incidence by age group, annual rates generally decreased as age increased, with the notable exception of 2017 and 2018, when the incidences among 20–39-year-olds and 40–59-year-olds exceeded those of younger age groups, which were less than 1 per 100,000. Among those aged 20–39 years, the annual incidence in 2017 stood out at over 6 per 100,000 (Figure 2B).

**Figure 2:**
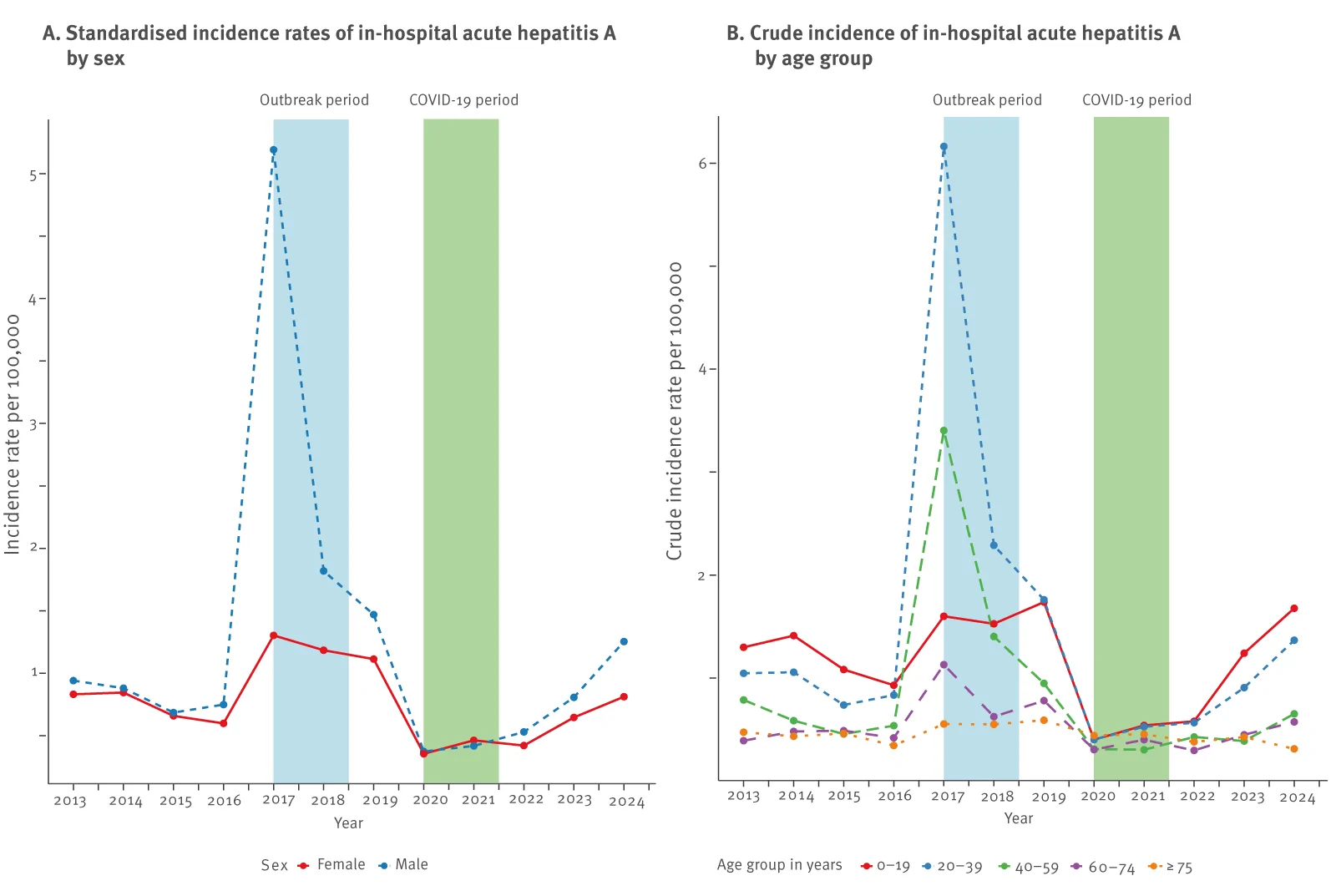
Incidence rates of hepatitis A cases in hospitals per 100,000 population by (A) sex (standardised) and (B) age group (crude), France, 2013–2024 (n = 7,923 inpatients)

The data concern France, including Metropolitan France and Overseas Departments.

Patient characteristics differed particularly between B and D periods (Table 1). During period B, most patients were men (76.22%), with a high prevalence of HIV infection (10.01%), AUD (8.06%) and smoking habits (11.33%), and a low prevalence of risk factors for chronic liver disease (3.47%), cirrhosis (1.78%), type-2 diabetes (3.89%), obesity (7.73%), severe comorbidities (7.78%), and deprivation (35.47%). By contrast, during period D, most patients were women (53.38%) and had a high prevalence of risk factors for chronic liver disease (4.83%), cirrhosis (3.14%), diabetes (7%), obesity (11.35%), and of CCI ≥ 3 (25.36%).

The proportion of hard outcomes also differed significantly between periods (Table 1). Case fatality rate was lowest during the outbreak in MSM (0.62%) and highest during the COVID-19 pandemic (2.90%), while the proportion of severe cases was lowest in 2013−2016 (22.85%) and highest during the outbreak in MSM (33.29%). In addition, a trend analysis showed a non-significant increase in the age- and sex-standardised proportion of severe HA over time (p = 0.065, Figure 1). The higher risk of severe disease in the most recent period was instead established by the multivariable and propensity-matched models (Table 2). Of note, as shown in Table 1, the most recent period (period E) ranked second for the proportion of men (57.15%), of severe comorbidities (14.18%), and for the case-fatality rate (2.32%).

**Table 2:** Multivariate analysis of risks associated with severity and death in patients hospitalised for HA, France, 2013–2024 (n = 7,923 inpatients)

Characteristic		Severe hepatitis A		Hepatitis A-related death	
		aOR: (95% CI)	p value	aOR: (95% CI)	p value
**Age in years, per decade over 30 years**		1.15 (1.11–1.20)	< 0.001	1.24 (1.07–1.44)	0.005
**Male sex**		1.38 (1.24–1.54)	< 0.001	1.29 (0.83–2.01)	0.27
**Smoking habits**		1.23 (1.02–1.48)	0.031	0.94 (0.48–1.73)	0.85
**Alcohol use disorders**		1.13 (0.89–1.42)	0.32	1.62 (0.79–3.17)	0.17
**Obesity^a^**		1.01 (0.83–1.22)	0.94	1.61 (0.91–2.73)	0.090
**Type-2 diabetes mellitus**		1.00 (0.78–1.27)	0.98	0.77 (0.42–1.34)	0.37
**Liver risk factors^b^**		1.29 (1.01–1.65)	0.041	0.50 (0.20–1.10)	0.11
**Cirrhosis**		1.40 (0.99–1.98)	0.054	2.90 (1.38–5.91)	0.004
**HIV infection**		1.22 (0.94–1.56)	0.13	0.20 (0.01–1.03)	0.13
**CCI ≥ 3**		0.68 (0.56–0.83)	< 0.001	12.3 (6.11–25.2)	< 0.001
**Fdep ≥ Q4**		0.94 (0.84–1.04)	0.21	1.21 (0.80–1.84)	0.36
**Observation period**	A: Jan 2013–Dec 2016	Reference		Reference	
	B: Jan 2017–Jun 2018	1.52 (1.32–1.75)	< 0.001	0.85 (0.42–1.70)	0.66
	C: Jul 2018–Dec 2019	1.46 (1.25–1.71)	< 0.001	0.96 (0.46–1.91)	0.90
	D: Jan 2020–Jun 2021	1.21 (0.94–1.55)	0.13	1.36 (0.62–2.84)	0.43
	E: Jul 2021–Dec 2024	1.46 (1.26–1.70)	< 0.001	1.86 (1.07–3.30)	0.031

aOR: adjusted odds ratio; CCI: Charlson Comorbidity Index; CI: confidence interval; Fdep: French Deprivation Index; HIV: human immunodeficiency virus.

^a^ Body mass index > 30 kg/m^2^.

^b^ These are risk factors for chronic liver disease, as detailed in the Methods section.

### Factors associated with severe hepatitis A in hospitals

Severe HA was identified in 2,277 patients (28.7%), and was associated with age, sex, smoking, AUD, diabetes, risk factors for chronic liver disease, cirrhosis, HIV, Fdep, and the predefined study periods covering the outbreak in MSM and the COVID-19 pandemic, but not obesity or CCI, as detailed in Supplementary Table S2.

In a multivariate analysis, independent risk factors for severe HA (Table 2) were advanced age (+ 15% of risk per decade over 30 years, p < 0.001), male sex (+ 38%, p < 0.001), smoking (+ 23%, p = 0.031), risk factors for chronic liver diseases (+ 29%, p = 0.041), and, at the limit of significance, cirrhosis (+ 40%, p = 0.054). A CCI ≥ 3 was negatively associated with severe HA (aOR: 0.68, p < 0.001), suggesting differences in the provision of care for patients with significant comorbidities, as the ICD-10 codes used to define severity include both diagnosis codes corresponding to organ failure (probably occurring in these frail patients) and codes corresponding to organ support therapeutic procedures. Compared with the reference period A, the risk of severe HA increased by 52%, 46%, and 46% for periods B, C, and E, respectively (p < 0.001; Table 2).

After adjustment for all covariates using propensity score matching, the risk of severe HA increased by 42% (aOR: 1.42; 95% CI: 1.24–1.63, p < 0.001), 49% (aOR: 1.49; 95% CI: 1.27–1.75, p < 0.001), 28% (aOR: 1.28; 95% CI: 1.00–1.64, p = 0.054), and 57% (aOR: 1.57; 95% CI: 1.35–1.83, p < 0.001), during periods B, C, D and E, respectively, as described in Supplementary Table S3, with Figure S3-A, showing adequate covariate balancing after matching. 

Excluding the CCI from the models did not alter the temporal trend. In the multivariable analysis, the aORs for severe HA in 2021–2024 compared to 2013–2016 were 1.46 (95% CI: 1.26–1.70) with the CCI vs 1.44 (95% CI: 1.23–1.67) without it; in the propensity-matched analysis the corresponding estimates were 1.57 (p < 0.001) vs 1.41 (p < 0.001). In the multivariable analysis, other risk factors were essentially unchanged, apart the loss of association with risk factors for chronic liver disease and cirrhosis, as described in Supplementary Table S4.

We then examined the mandatory notification data, which also indicate whether a reported case is hospitalised or not, to determine how the hospitalisation rate among reported cases had changed, hospitalisation being the marker of a particularly symptomatic or even severe infection. Mean hospitalisation rate was 51% but we observed a significant and steady increase in the proportion of hospitalised cases among those reported from 2013 onwards (p = 0.004 for the trend, Figure 3).

**Figure 3:**
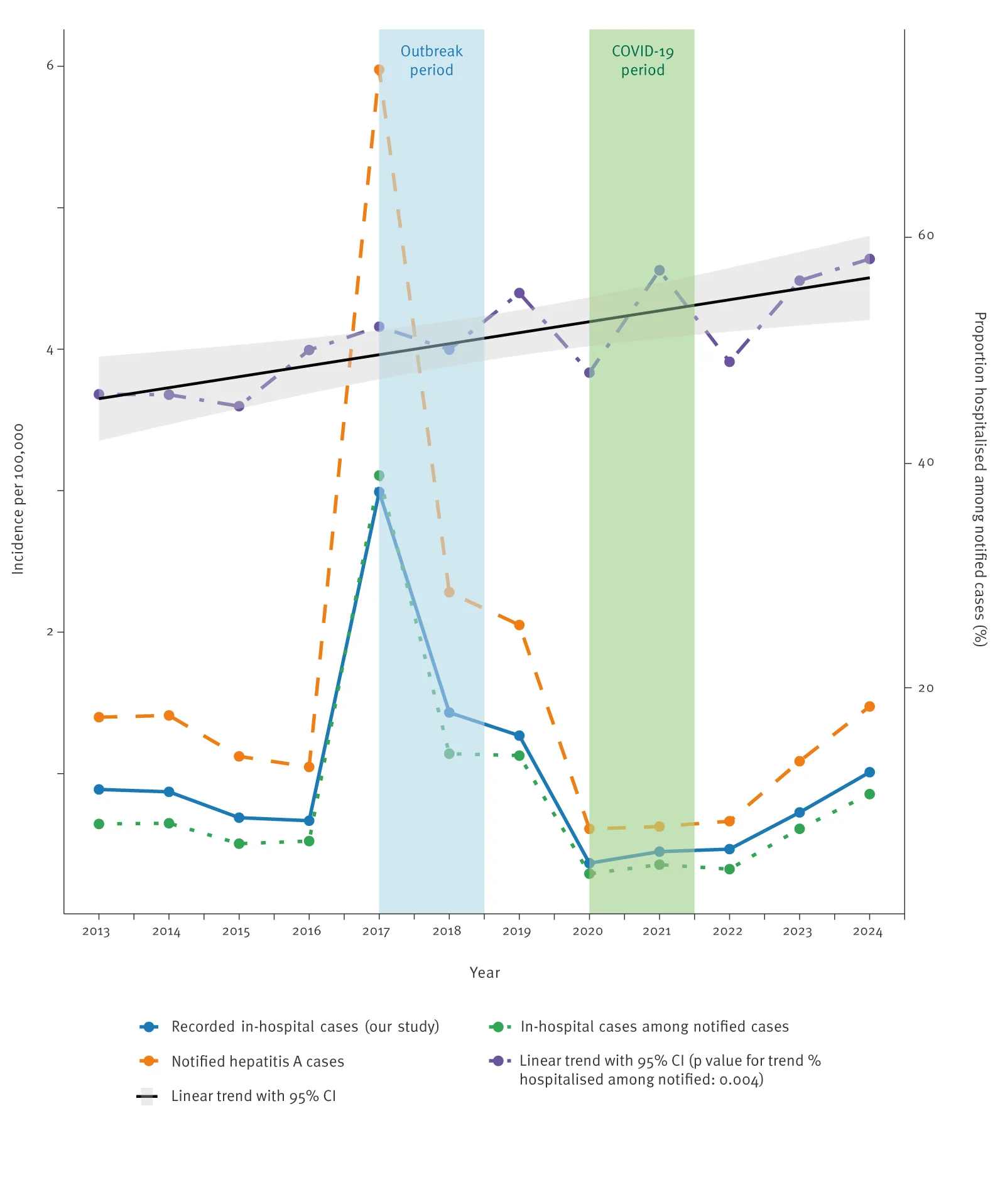
Crude incidences per 100,000 population of hepatitis A cases in the general population (mandatory reporting data) and in hospitals (National Hospital Discharge Data Set), as well as proportion of cases hospitalised among notified cases, France, 2013–2024 (notified cases n = 13,226; hospitalised cases n = 7,923 inpatients)

The data concern France, including Metropolitan France and Overseas Departments.

Data from mandatory reporting include incidence of notified hepatitis A cases in the general population (dashed orange line), of hospitalisation among notified cases (dotted green line), proportion of hospitalised cases among notified cases (dotted/dashed purple line), and the linear trend of hospitalisation proportion among notified cases (black line; p = 0.004). Incidence of hepatitis A cases in French hospitals (blue line) shows similar trends to other incidences.

### Factors associated with hepatitis A-related death in hospitals, France, 2013–2024

In-hospital death was recorded for 103 patients, corresponding to an overall case-fatality rate of 1.30% (Table 1). Factors associated with death were age, AUD, obesity, diabetes, cirrhosis, CCI, and predefined study periods but not sex, smoking, risk factors for chronic liver disease, HIV, or Fdep (Supplementary Table S2).

In a multivariate analysis (Table 2), independent risk factors for death were age (aOR: 1.24, p = 0.005), cirrhosis (aOR: 2.90, p = 0.004), and CCI ≥ 3 (aOR: 12.3, p < 0.001). The risk of death did not vary significantly during the first four periods. However, compared with the reference period, this risk was nearly twice as high in the fifth period, 2021–2024 (aOR: 1.86, p = 0.031) (Table 2). This result was confirmed after propensity score matching (Supplementary Table S3 and Figure S3-B). After adjustment for all covariates, the probability of death in 2021–2024 remained nearly twice that of the reference period (aOR: 1.96; 95% CI: 1.13–3.40; p = 0.016). 

Excluding the CCI did not alter the temporal trend: compared to 2013–2016, aORs for death in 2021–2024 were 1.86 (95% CI: 1.07–3.30) with the CCI vs 2.15 (95% CI: 1.25–3.77) without it, in the multivariable analysis, and 1.96 (95% CI: 1.14–3.45; p = 0.016) vs 1.81 (95% CI: 1.07–3.12; p = 0.030) after matching. Regarding risk factors in the multivariable analysis, excluding the CCI strengthened the association with age (aOR: 1.24, 95% CI: 1.07–1.44 vs 1.90, 95% CI: 1.70–2.13; without CCI) and with cirrhosis (aOR: 2.90, 95% CI: 1.38–5.91 vs 3.64; 95% CI: 1.77–7.27 vs 2.90) and revealed an association with obesity (aOR: 1.87; 95% CI: 1.07–3.13, p = 0.022) (Supplementary Table S4).

## Discussion

Our retrospective study of nearly 8,000 HA-related hospitalisations in France over 12 years covered two major events: the epidemic in MSM in 2017–2018 [8], when incidence of HA in hospitals peaked, and the COVID-19 pandemic in 2020–2021 [18], when it was lowest. Overall, 28.7% of hospitalised patients developed severe HA, and 1.30% died. This case fatality rate is consistent with the 1.30% rate reported in France during 2008–2013 [22]. As others have noted, determinants of severity and mortality differ [23-27]. In our cohort, age, male sex, risk factors for chronic liver disease, cirrhosis, and smoking increased the risk of severe disease, while age, cirrhosis and CCI > 3 predicted death. However, in models excluding the CCI — which is closely related to age — the association with age increased moderately*,* suggesting that the excess mortality of older patients is largely mediated by their comorbidity burden rather than by age itself. That fatal outcomes are driven by comorbidities is in keeping with the equitable hospital access available in France and with the absence of any association between social vulnerability and outcomes. Our finding may reflect limitations using organ support procedures in patients with poor physiological condition [17]. Notably, we observed an increased risk for severe disease and a near-doubling of the risk of hospital mortality from mid-2021 onwards compared with pre-2017, a trend also reflected by the rise in hospitalisation rate among cases reported through mandatory notification. To our knowledge, such progression had not been prior documented.

Previous studies in high-income countries have analysed HA-related hospitalisations and their evolution over time. Those performed from the late 1990s to mid-2010s in periods of low and declining HA incidence in the United States (US), Taiwan, and Europe, found decreasing incidence and no increase in severity or mortality, despite rising age and comorbidities [23-25,28]. More recent studies, including ours, spanned major HA epidemics. An American study concerning the US (1998–2020) [26] covered HA outbreaks among people who use drugs and people who experience homelessness from 2016 onward, and a Spanish study (2000–2021) [27] included the 2016–2018 European HA epidemic in MSM [8]. Both reported sharp increases of HA incidence in hospitals during HA epidemics, followed by declines during the COVID-19 pandemic. The data from the US showed no significant change in HA-related hospital mortality, which averaged 2.7% [26], despite high hospitalisation frequency (up to 84.8%) and variable case fatality (0–10.8%) shown in a systematic review conducted to assess outcomes among outbreak-associated cases [29]. The Spanish study reported no trend analysis of mortality but a low mortality rate (0.3%), likely reflecting the epidemics in MSM, in which cases presented very low case fatality (0.03–0.26%) [7]. Neither study analysed changes in severity, or patient characteristics across epidemic and non-epidemic periods.

In France, surveillance data based on mandatory reporting have shown substantial variation in age and sex distribution depending on viral circulation and epidemic contexts. Accordingly, to analyse temporal trends in the current study, we defined time frames accounting for the HA outbreak in MSM and the COVID-19 pandemic. This allowed us to detect considerable differences in cases’ age, sex, and comorbidities between these time frames, as well as variations in the case fatality rate, which was lowest in 2017–2018 (0.6%), during the HA epidemic in MSM [7], and highest during 2020–2021 (2.9%), possibly reflecting pressure on the healthcare system during the COVID-19 pandemic, as also suggested by the US study [26]. Regarding clinical severity, comparison between studies is challenging because definitions vary. We defined severity by ICD-10 codes corresponding to diagnoses of organ failures and therapeutic procedures for organ support, including LT — an outcome-based, objective measure, validated in previous studies [12,30]. Indicators such as prolonged hospital stay, often used as proxies, may reflect specific organisational factors [25] and are thus less reliable. 

The HA incidence was higher among men than women throughout the study, particularly in 2017, when the difference was fourfold. This period, coinciding with the HA epidemic in MSM, also marked a shift in the age categories affected by HA, with the highest incidence in the 20–39-year and the 40–59-year age groups; this was consistent with data from the epidemic in MSM, showing a median age of 35 years [7]. It is likely that this shift towards older ages contributed to the highest severity rate of all periods, i.e. 33%. While most comorbidities, particularly liver disease and cirrhosis, were less common among cases occurring during this period than in other periods, smoking and HIV infection were more frequent. However, in our study, HIV infection did not increase the risk of HA severity or mortality, consistent with findings from a large cohort showing a prolonged but milder course of disease [31]. On the other hand, smoking correlated with severity but not with death. Smoking is strongly linked to poor outcomes in chronic liver disease [32] and, although HA is self-limiting, smoking-related immune impairment could heighten risk of organ failure. Probably because young, otherwise healthy smokers can recover from liver failure with appropriate management, we did not find an association with increased mortality risk. However, unlike results from Wasuwanich et al. [26], smoking was not associated with reduced mortality in the current study.

A major finding was the apparent increase of HA severity over time, evidenced by the increase in hospitalisation rates among notified cases from 2013 onwards and higher severity levels among hospitalised cases in recent years, coinciding with a resurgence of viral circulation, linked to renewed travel and loosening of preventive measures after the COVID-19 pandemic. Two complementary mechanisms may account for this trend. First, the population hospitalised for HA has shifted towards older, more frequently male, and more comorbid patients, factors associated with poor outcomes. Second, the increase in the risk of both severe disease and death persisted after propensity-score matching for confounders, (i.e. identified risk factors for severity and death), and was unchanged when the CCI was excluded from the models, indicating an effect not fully explained by the measured case-mix. One unlikely hypothesis is changes in viral virulence, as proposed for recent US epidemics [26,33]. Indeed, genetic determinants for adaptation to cell culture and pathogenic phenotype (i.e. virulence/attenuation) have been identified in HAV and these have been exploited during live vaccine development [34]. Some studies also suggest that nucleotide variations-or specific genotypes may correlate with higher aminotransferase levels [1], though this reflects acute liver injury and does not equate to clinical severity. Given HAV’s non-cytopathic nature and complex immune-mediated pathogenesis [35], viral genetics likely play a modest role compared with host factors. In addition, the French Reference Centre surveillance revealed no major genotype shifts and high strain diversity since the 2010s, arguing against emergence of a virulent variant. The observed increase in severity and mortality thus remains unexplained and warrants further investigation, including potential cognitive biases in clinical decision-making [36]. It is conceivable that, as was the case during the COVID-19 period, when care resources were not prioritised for the oldest patients, resources allocated to the treatment of HA may have been focused more on younger MSM — perceived at higher risk — while older patients with multiple comorbidities received less intensive management.

Administrative databases concerning hospital discharge information provide large, multicentric, and diverse cohorts but have limitations. Firstly, patient-level data are not available. Secondly, outpatient cases are not captured, so findings apply to hospitalised patients only. Thirdly, reliance on coded diagnoses and procedures may lack both sensitivity and specificity, as their accuracy depends on clinicians’ coding practices. Diagnosis of HA, based on anti-HAV immunoglobulin (Ig)M detection, is generally straightforward but still subject to miscoding or false-positive IgM results, potentially inflating case counts [25,37]. To improve specificity, we excluded patients where HA appeared as an associated diagnosis, however, some patients with jaundice, acute liver failure or decompensated cirrhosis who were excluded may have been true cases. However, our trends and case numbers aligned with national surveillance data, validating our selection approach. In addition, coding of our chosen outcomes (mortality and severity) has demonstrated 100% accuracy in prior French studies [12,30]. Therefore, our approach to select patients likely ensured high specificity, but at possible cost of a loss of sensitivity. Finally, mortality estimates rest on a limited number of deaths (n = 103), which widens the CIs and calls for confirmation in other settings.

Safe, highly immunogenic vaccines for HA have been available since the early 1990s, providing both pre- and post-exposure protection. In France, consistent with World Health Organization guidance for low-endemicity areas, vaccination targets groups at increased infection risk (travellers, MSM) or severe-disease risk (chronic liver disease) [38]. French vaccination policy also includes children of immigrant parents and patients with cystic fibrosis (https://sante.gouv.fr/prevention-en-sante/preserver-sa-sante/vaccination/calendrier-vaccinal). However, it excludes migrants, people in prison, people who experience homelessness, and people who use drugs, among whom seroprevalence is decreasing, as in the general population [39]. Moreover, outbreaks among MSM demonstrated under-vaccination even within targeted groups [40]. Declining seroprevalence and recurring epidemics among adults argue for strengthening the current targeted strategy and possibly extending vaccination to the general population, regardless of whether or not there is an identified risk factor [41].

## Conclusion

This national study provides the first comprehensive analysis of HA prognosis among hospitalised patients in France, where universal access minimises selection bias. Age, male sex, chronic liver disease, cirrhosis, and smoking increased the risk of severe HA, while cirrhosis and high-CCI — the latter also accounting for the effect of age —predicted HA-related death. Both severity and mortality of HA in hospitals rose in recent years, an increase that persisted after adjustment for, and matching on, comorbidities, probably driven by a shift towards older, multimorbid patients. Crucially, these outcomes are vaccine-preventable, and most patients who die today have conditions for which vaccination is currently recommended (or very similar conditions), yet remain under-immunised, as others have pointed out. Our findings warrant confirmation in other low-endemicity countries to inform future vaccination strategies, potentially supporting broader or universal vaccination policies.

## Data Availability

Code availability: The analysis code is openly available on GitHub (https://github.com/demosthenes-group/hav_2013_2024) and archived on Zenodo (https://doi.org/10.5281/zenodo.21610784). The repository includes the full R pipeline and the ICD-10/CCAM code dictionary. The PMSI hospital-discharge data are protected health data (CNIL authorisation DR-2017-404) and cannot be redistributed; access can be requested through the French Health Data Hub.

## Preprint

This work was published as a preprint article on MedRxiv (Roque-Afonso AM, Mouliade C, Parlati L, Goutté N, Figoni J, Bouam S, et al. Trends and risk factors associated with severity and mortality related to hepatitis A in French hospitals: a national population-based study, 2013 to 2024). https://doi.org/10.64898/2026.04.30.26351819

## Supplementary Data

716000 SupplementaryMaterial
