# Supplementary material for: Trends and risk factors associated with hepatitis A severity and mortality in French hospitals: a national population-based study, 2013 to 2024
Source: Euro Surveill. 2026 Oct 1;31(39):2500858. doi: 10.2807/1560-7917.ES.2026.31.39.2500858 (PMC13633467; doi:10.2807/1560-7917.ES.2026.31.39.2500858)

SUPPLEMENTARY MATERIAL (updated)

Trends and risk factors associated with hepatitis A severity and mortality in French hospitals: a national population-based study, 2013 to 2024

This supplementary material is hosted by *Eurosurveillance* as supporting information alongside the article "Trends and risk factors associated with hepatitis A severity and mortality in French hospitals: a national population-based study, 2013 to 2024", on behalf of the authors, who remain responsible for the accuracy and appropriateness of the content.

The same standards for ethics, copyright, attributions and permissions as for the article apply.

Supplements are not edited by *Eurosurveillance* and the journal is not responsible for the maintenance of any links or email addresses provided therein.

Contents:

Supplementary Methods

Supplementary Tables

- **Table S1:** ICD-10 / CCAM code dictionary
- **Table S2:** Characteristics of patients by hepatitis A severity and mortality
- **Table S3:** Propensity-score matched models for severe hepatitis A and death
- **Table S4:** Sensitivity analysis with and without the Charlson Comorbidity Index

Supplementary Figures

- **Figure S1: Study Flowchart**
- **Figure S2: Quarterly number of HA cases recorded in hospitals (total, severe and deaths), France, 2013-2024, n=7,923 inpatients**
- **Figure S3: Covariate balance after propensity score matching for severe Hepatitis A (A) and Hepatitis A-Related Death (B), French Hospitals, 2013-2024**

Supplementary Methods

Additional information on PMSI data source

The French Hospital Discharge Database coding system has demonstrated high validity for hard outcomes requiring hospital care. In a previously published nationwide study using the same database and analytical framework, liver-related outcomes were defined as hospital-managed events, including decompensated cirrhosis, primary liver cancer related to cirrhosis, and liver transplantation, the latter being exhaustively identified through the national transplantation registry. Mortality was assessed using in-hospital death records. In that study, a sensitivity analysis was conducted to estimate mortality occurring outside hospital, based on the morbidity profile of patients discharged with chronic liver disease and subsequently lost to follow-up. This analysis showed that in-hospital deaths accounted for 97.0% of overall mortality (95% CI: 96.6–97.3). Analyses using overall mortality instead of in-hospital mortality yielded similar results, supporting the use of in-hospital mortality and hospital-recorded liver-related outcomes as reliable endpoints in this database [Schwarzinger et al. 2017].

Data analysis: calculation of standardized incidence rates

Standardized rates were computed by direct standardization using the European Standard Population 2013 (ESP2013) [18] as the reference, with five age groups - 0–19, 20–39, 40–59, 60–74, and 75 years or older - weights 0.214, 0.272, 0.291, 0.149 and 0.074, respectively, summing to 1. For the overall standardised rate, sex-specific case and population counts were pooled within each age stratum before the ESP2013 age weights were applied, so that the age weights are applied once and are not double-counted across sexes; the sex-specific curves are age-standardised within each sex. Severe HA standardized rates were derived using the identical procedure, restricted to hospitalizations meeting the severity definition. The comparison between notified (mandatory-reporting) and hospitalised incidence uses crude rates throughout, to match the mandatory-notification data, which are available as crude counts only

Supplementary Tables

Table S1: ICD10 code dictionary.

Highlighted in yellow are the codes used to define severe Hepatitis A: i.e. hepatic and/or extrahepatic organ failure-within 12 weeks of admission with the first record of HA as primary discharge diagnosis. These codes correspond to diagnosis codes of organ failure and to organ support therapeutic procedures, including LT.

Diagnosis	ICD-10/Medical Procedure Codes
Acute and Subacute Hepatic Failure	K72.0, K71.2
Acute Hepatitis A with Hepatic Coma	B15.0
Acute Hepatitis A without Hepatic Coma	B15.9
Acute Kidney Injury	N17-, JVJF002, JVJF003, JVJF005, JVJF006, JVJF007, JVJF005, JVJB002
Acute Respiratory Failure	J96.0, J80-, J81-, GLLD002, GLLD003, GLLD017, GLLD019, GLLD004, GLLD005, GLLD006, GLLD007, GLLD008, GLLD009, GLLD012, GLLD013, GLLD015, DKMD001, GEPA004, GELD002, GELD004, ECCO2R, GLJF010
AIDS	B20-, B21-, B22-, B24-
Alcohol Rehabilitation	Z50.2, Z71.4
Alcohol Use Disorders	E24.4, E51.1, F10.1, F10.2, F10.3, F10.4, F10.5, F10.6, F10.7, F10.8, F10.9, F10.2, F10.2, F10.2, F10.2, G31.2, G62.1, G72.1, I42.6, K29.2, K70-, K85.2, K86.0, O35.4, Z50.2, Z71.4, Z72.1
Alcoholic Cirrhosis	K70.3
Alcoholic Hepatic Failure	K70.4
Alcoholic Hepatitis	K70.1
Alcoholic Liver Disease	K70-
Ascitis	R18-, HPHB003, HPJB001
Aspergillosis	B44-
Autoimmune Hepatitis	K75.4
Bacteremia	A49-

Diagnosis	ICD-10/Medical Procedure Codes
Bronchopneumonia	J18-
Budd Chiari Syndrome	I82.0
Cerebral Infarction	I63-
Cerebrovascular Disease	G45-, G46-, H34.0, I6
Chemotherapy for Cancer	Z51.1
Chemotherapy for Connective Tissue Disorder	Z51.2
Cholangitis	K83.0, K83.1, K83.5, K83.5, K87.0, R17-
Chronic Hepatitis B	B18.1, B16.2, B16.9
Chronic Hepatitis C	B18.2
Chronic Hepatitis D	B16.0, B16.1, B18.0, B17.0
Chronic Kidney Disease Advanced	I12.0, I13.1, N00-, N01-, N03-, N05-, N18.3, N18.4, N18.5, N19-, N25.0, Z49.0, Z49.1, Z49.2, Z94.0, Z99.2, HGPC005, HPGA001, HPJP001, HPKA002, HPKB001, HPKC014, HPLA005, HPLB004, HPLC035, HPPA004, HPPP002, JVRP007, JVRP008, JAEA003, JVJB001, JVRP004, JVJF004, JVJF008, JVQF001, JVQF007, JVQP002, JVQP009, YYYY007
Chronic Kidney Disease	E10.2, E11.2, E12.2, E13.2, E14.2, I15.1, JAHB001, JAHH002, JAHC001, JAHA001, JAHJ006, JAHJ007, N02-, N04-, N06-, N07-, N08-, N18.1, N18.2, N19-, N25-, N08.3
Chronic Obstructive Pulmonary Disease	I27.8, I27.9, J40-, J41-, J42-, J43-, J44-, J45-, J46-, J47-, J60-, J61-, J62-, J63-, J64-, J65-, J66-, J67-, J68.4, J70.1, J70.3
Cirrhosis	I85.9, I86.4, I98.2, I98.2, K70.3, K71.7, K74.3, K74.4, K74.5, K74.6, K76.6
Congestive Heart Failure	I09.9, I11.0, I13.0, I13.2, I25.5, I42.0, I42.5, I42.6, I42.7, I42.8, I42.9, I43-, I50-, P29.0
Connective Tissue Disorder	M05-, M06-, M31.5, M32-, M33-, M34-, M35.1, M35.3, M36.0
Cryptococcosis	B45.0, B45.1, B45.3, B45.7
Decompensated Cirrhosis	R17-, K70.4, K71.1, K72-, K76.7, I28.0, I85.0, I98.3, I98.2, R18-, J94.8, K65-, EHBD001, EHNE002, HESE001, HESE002, HPHB003, HPJB001, EHCA003, EHCA006, EHCA009, EHCA007, EHCA004, EHCA002, EHCA005, EHCA010, EHCA001, HEPA005, HEPA004, HEPA007

Diagnosis	ICD-10/Medical Procedure Codes
Dementia	F00-, F01-, F02-, F03-, F05.1, G30-, G31.1
Diabetes Mellitus Complicated	E10.2, E10.3, E10.4, E10.5, E10.7, E11.2, E11.3, E11.4, E11.5, E11.7, E12.2, E12.3, E12.4, E12.5, E12.7, E13.2, E13.3, E13.4, E13.5, E13.7, E14.2, E14.3, E14.4, E14.5, E14.7, H36.0, N08.3, H28.0, G63.2
Diabetes Mellitus Uncomplicated	E10.0, E10.1, E10.6, E10.8, E10.9, E11.0, E11.1, E11.6, E11.8, E11.9, E12.0, E12.1, E12.6, E12.8, E12.9, E13.0, E13.1, E13.6, E13.8, E13.9, E14.0, E14.1, E14.6, E14.8, E14.9
Diabetes Mellitus	E1
Disseminated Intravascular Coagulation	D65-, D68.4
Extracorporeal Co 2 Removal	ECCO2R, GLJF010
Embolism and Thrombosis of Renal Vein	I82.8
Embolism and Thrombosis of Unspecified Vein	I82.9
Embolism and Thrombosis of Vena Cava	I82.2
Gastro Esophageal Varices Bleeding	I85.0, I98.3, I98.2, EHBD001, EHNE002, HESE001, HESE002
Gastro Esophageal Varices not Bleeding	I85.9, I86.4, I98.2, I98.2
Hemiplegia	G04.1, G11.4, G80.1, G80.2, G81-, G82-, G83.0, G83.1, G83.2, G83.3, G83.4, G83.9
Hepatic Encephalopathy	K72.0, G94.3, R40.2
Hepatocellular Carcinoma Treatment	YYYY170, EDLF017, EDLF016, HLNM001, EDQH006, EDLF014, YYYY210, HLHJ005, EDQH007, HLHJ006, EDLF015, EDQH008, HLNK001, EDSF006, EDLL001, ZZQA002, EHSF001, HLQX004, EDLL002, HLFA020
Hepatocellular Carcinoma	C22.0
Hepatopulmonary Syndrome	I28.0
Hepatorenal Syndrome	K76.7
HIV Infection	Z21-, B20-, B21-, B22-, B23-, B24-

**Diagnosis**	**ICD-10/Medical Procedure Codes**
Hydrothorax	J94.8
Hypercholesterolemia Pure	E87.0
Hyperlipemia Mixed	E78.2
Hyperlipemia	E78.0, E78.1, E78.2, E78.4, E78.5
Hypertension	I10-, I11-, I12-, I13-, R03.0
Hypertensive Heart Failure with or without Renal Failure	I10-, I13.0, I13.2
Hypertriglyceridemia	E87.1
Intrahepatic Bile Duct Carcinoma	C22.1
Invasive Candidiasis	B37.1, B37.5, B37.6, B37.7
Jaundice	R17-
Leukemia	C91-, C92-, C93-, C94-, C95-, C96-
Liver Biopsy	HLHB001, HLHJ003, HLHJ006, HLHH006, HLHJ005, HLHH007, HLHH001, HLHH005
Liver Disease Mild	K71.3, K71.4, K71.5, K73-, K74.0, K74.1, K74.2, K74.3, K76.0, K76.4, K76.5
Liver Disease Moderate to Severe	K70.3, I85.0, I85.9, I86.4, I98.2, J94.8, K70.3, K70.4, K71.1, K71.7, K72.1, K72.9, K74.4, K74.5, K74.6, K65-, K66-, K76.7, R17-, R18-, EHBD001, EHNE002, HESE001, HESE002, HPHB003, HPJB001, EHCA003, EHCA006, EHCA009, EHCA007, EHCA004, EHCA002, EHCA005, EHCA010, EHCA001, HEPA005, HEPA004, HEPA007
Liver Transplantation	HLEA001, HGEA002, HLEA002, HGEA004
Lymphoma	C81-, C82-, C83-, C84-, C85-, C86-, C87-, C89-, C90-
Mechanical Ventilation	GLLD004, GLLD005, GLLD006, GLLD007, GLLD008, GLLD009, GLLD012, GLLD013, GLLD015, DKMD001, GEPA004, GELD002, GELD004
Myocardial Infarction	I21-, I22-, I25.2
Obesity	E66-
Other Cause of Chronic Liver Disease	E83.0, E83.1, FEJF003, E84.8, I82.0, K83.0, K74.3, K75.4, Q44.2, Q44.3, Q44.6

**Diagnosis**	**ICD-10/Medical Procedure Codes**
Other Diabetes Mellitus	E12-, E13-, E14-
Other Primary Liver Cancer	C22.2, C22.3, C22.4, C22.7, C22.9
Oxygen Therapy	GLLD002, GLLD003, GLLD017, GLLD019
Palliative Care	Z51.5
Paraplegia Hemiplegia	G04.1, G11.4, G80.1, G80.2, G81-, G82-, G83.0, G83.1, G83.2, G83.3, G83.4, G83.9
Peptic Ulcer Disease	K25-, K26-, K27-, K28-
Peritonitis	K65-
Peripheral Vascular Disease	I70-, I71-, I73.1, I73.8, I73.9, I77.1, I79.0, I79.2, K55.1, K55.8, K55.9, R02-, Z95.8, Z95.9
Phlebitis and Thrombophlebitis	I80-
Pneumocystosis	B59-
Pneumonia Bacterial	J13-, J14-, J15-, J16-, J17.0, J17.8, J18-, J85-
Pneumonia Due to Streptococcus Pneumoniae	J13-
Pneumonia Due to Haemophilus Influenzae	J14-
Pneumonia Bacterial not Elsewhere Classified	J15-
Pneumonia Due to Other Infectious Organisms not Elsewhere Classified	J16-
Pneumonia in Diseases Classified Elsewhere	J17.0, J17.8
Portal Hypertension	K76.6
Portal Vein Thrombosis	I81-
Renal Replacement Therapy	JVJF002, JVJF003, JVJF005, JVJF006, JVJF007, JVJF005, JVJB002
Renal Transplantation	T86.1, Z94.0, JAEA003
Shock	I46-, R57-, A48.3, EQLF001, EQLF003

Diagnosis	ICD-10/Medical Procedure Codes
Systemic Inflammatory Response Syndrome of Infectious Origin	A40-, A41-, A49-, R57.2, R65.0
Smoking	F17-, Z71.6, Z72.0, T65.2
Solid Tumor Localised without Liver	C0, C1, C20-, C21-, C23-, C25-, C26-, C3, C40-, C41-, C43-, C45-, C46-, C47-, C48-, C49-, C5, C6, C70-, C71-, C72-, C73-, C74-, C75-, C76-
Solid Tumor Localised	C0, C1, C20-, C21-, C22-, C23-, C24-, C25-, C26-, C3, C40-, C41-, C43-, C45-, C46-, C47-, C48-, C49-, C5, C6, C70-, C71-, C72-, C73-, C74-, C75-, C76-
Solid Tumor Metastatic	C77-, C78-, C79-, C80-
Splanchnic Thrombophlebitis	I81-, I82-
Surgical Shunt	EHCA003, EHCA006, EHCA009, EHCA007, EHCA004, EHCA002, EHCA005, EHCA010, EHCA001, HEPA005, HEPA004, HEPA007
Syphilis	A51-, A52-, A53-
Thrombophlebitis Migrans	I82.1
Transfusion	FELF003, FELF004
Transjugular Intrahepatic Posto Systemic Shunt	EHCF002, EHAF004, EHPF001, EHNF001
Transjugular Liver Biopsy	HLHH001, HLHH005
Transplant Recipient without Liver	Z94.0, Z94.1, Z94.2, Z94.3, Z94.8, Z94.8, T86.1, T86.2, T86.3, T86.8, T86.8, T86.8, Z94801, Z94802, Z94803, Z94804, Z94809, T86.0, JAEA003
Type 1 Diabetes Mellitus	E10-
Type 2 Diabetes Mellitus	E11-
Urinary Tract Infection	N10-, N39.0
Vasopressor	EQLF001, EQLF003

Table S2: Characteristics of patients hospitalized for hepatitis A according to severity and case-fatality. France, 2013-2024 (n=7,923 inpatients; univariate analysis)

Characteristic	Overall		Severe Hepatitis A					Hepatitis A-related death				
			No		Yes		p-value[1]	No		Yes		p-value[2]
	n	%	n	%	n	%		n	%	n	%	
	7,923	100	5,646	71.3	2,277	28.7		7,820	98.7	103	1.30	
**Age, Median (IQR)**	30.0 (18.0, 48.0)		28.0 (16.0, 47.0)		35.0 (23.0, 51.0)		<0.001	30.0 (18.0, 48.0)		73.0 (48.0, 85.0)		<0.001
**Male Sex**	4,805	60.65	3,271	57.93	1,534	67.37	<0.001	4,740	60.61	65	63.11	0.61
**Smoking Habits**	666	8.41	425	7.53	241	10.58	<0.001	651	8.32	15	14.56	0.023
**Alcohol Use Disorders**	462	5.83	284	5.03	178	7.82	<0.001	441	5.64	21	20.39	<0.001
**Obesity**	649	8.19	451	7.99	198	8.70	0.30	628	8.03	21	20.39	<0.001
**Type-2 Diabetes Mellitus**	389	4.91	258	4.57	131	5.75	0.027	369	4.72	20	19.42	<0.001
**Liver Risk Factors**	327	4.13	207	3.67	120	5.27	0.001	319	4.08	8	7.77	0.075
**Cirrhosis**	175	2.21	100	1.77	75	3.29	<0.001	157	2.01	18	17.48	<0.001
**HIV Infection**	308	3.89	194	3.44	114	5.01	0.001	307	3.93	1	0.97	0.19
**CCI ≥ 3**	893	11.27	612	10.84	281	12.34	0.056	814	10.41	79	76.70	<0.001
**Fdep ≥ Q4**	3,191	41.37	2,313	42.11	878	39.55	0.039	3,141	41.26	50	49.50	0.095
**Period**							<0.001					<0.001
**A**	2,105	27	1,624	29	481	21		2,081	27	24	23	
**B**	2,418	31	1,613	29	805	35		2,403	31	15	15	
**C**	1,350	17	950	17	400	18		1,336	17	14	14	
**D**	414	5.2	305	5.4	109	4.8		402	5.1	12	12	
**E**	1,636	21	1,154	20	482	21		1,598	20	38	37	

Severe HA was hepatic and/or extrahepatic organ failure-within 12 weeks of admission with the first record of HA as primary discharge diagnosis. HA-related death was death within 12 weeks post-admission. Periods were A) before the outbreak among MSM; B) MSM outbreak; C) after outbreak until COVID-19 pandemic; D) COVID-19 pandemic, as defined for France; and E) after COVID-19 pandemic.

[1] Wilcoxon rank sum test; Pearson's Chi-squared test;

[2] Wilcoxon rank sum test; Fisher's Exact Test for Count Data with simulated p-value (based on 2000 replicates)

Table S3: Likelihood of Severe Hepatitis A and Hepatitis A-related Death in Propensity-Matched Patients in hospitals, France, 2013-2024

Characteristic	Severe HA<br>OR (95% CI)[1]	p-value	HA-related death<br>OR (95% CI)[1]	p-value
**Age (per decade > 30y)**	1.00 (1.00 to 1.00)	0.31	1.00 (0.99 to 1.01)	0.75
**Male Sex**	0.96 (0.86 to 1.07)	0.48	0.96 (0.62 to 1.50)	0.86
**Smoking Habits**	0.97 (0.80 to 1.14)	0.64	0.82 (0.41 to 1.52)	0.54
**Alcohol Use Disorders**	0.90 (0.72 to 1.12)	0.34	0.99 (0.48 to 1.91)	0.97
**Type-2 Diabetes Mellitus**	1.01 (0.80 to 1.27)	0.93	1.26 (0.68 to 2.22)	0.44
**Liver Risk Factors**	0.99 (0.78 to 1.24)	0.91	0.91 (0.37 to 1.91)	0.82
**Obesity**	0.94 (0.78 to 1.13)	0.53	0.89 (0.50 to 1.51)	0.67
**Cirrhosis**	1.18 (0.86 to 1.61)	0.29	1.24 (0.56 to 2.59)	0.58
**HIV**	1.01 (0.79 to 1.27°	0.96	0.70 (0.04 to 3.47)	0.73
**CCI ≥ 3**	0.81 (0.67 to 0.97)	0.021	0.85 (0.42 to 1.73)	0.65
**Fdep Q4 or Q5**	1.01 (0.91 to 1.12)	0.85	1.01 (0.67 to 1.52)	0.96
**Period**<br>**A [2013-2017[**	Reference		Reference	
**B [2017-Jun. 2018[**	1.42 (1.24 to 1.64)	<0.001	0.78 (0.38 to 1.54)	0.48
**C [Jul. 2018-2019]**	1.49 (1.27 to 1.75)	<0.001	0.98 (0.49 to 1.91)	0.96
**D [2020-Jun. 2021]**	1.28 (0.99 to 1.63)	0.054	1.03 (0.43 to 2.26)	0.94
**E [Jul. 2021-2024]**	1.57 (1.35 to 1.83)	<0.001	1.96 (1.14 to 3.45)	0.016

Risks were assessed using multivariate logistic regression models in propensity-matched samples (see figures S5 and S6 for covariates balances) adjusted for all studied variables.

Severe HA was hepatic and/or extrahepatic organ failure-within 12 weeks of admission with the first record of HA as primary discharge diagnosis. HA-related death was death within 12 weeks post-admission.

[1] OR = Odds Ratio, CI = Confidence Interval

Table S4: Sensitivity analysis: models with vs without the Charlson Comorbidity Index (CCI) for unmatched multivariable models (A) and propensity-score matched models (B)

A) Unmatched multivariable models

	Severe HAV (with CCI)		Severe HAV (w/o CCI)		HAV death (with CCI)		HAV death (w/o CCI)	
**Characteristic**	**OR (95% CI)**	**p-value**	**OR (95% CI)**	**p-value**	**OR (95% CI)**	**p-value**	**OR (95% CI)**	**p-value**
Age per decade over >30	1.15 (1.11 to 1.20)	<0.001	1.11 (1.07 to 1.14)	<0.001	1.24 (1.07 to 1.44)	0.005	1.90 (1.70 to 2.13)	<0.001
Male Sex	1.38 (1.24 to 1.54)	<0.001	1.38 (1.24 to 1.54)	<0.001	1.29 (0.83 to 2.01)	0.27	1.25 (0.81 to 1.95)	0.32
Smoking Habits	1.23 (1.02 to 1.48)	0.031	1.21 (1.01 to 1.46)	0.042	0.94 (0.48 to 1.73)	0.85	1.10 (0.57 to 2.01)	0.77
Alcohol Use Disorders	1.13 (0.89 to 1.42)	0.32	1.12 (0.89 to 1.41)	0.33	1.62 (0.79 to 3.17)	0.17	1.78 (0.89 to 3.43)	0.093
Obesity	1.01 (0.83 to 1.22)	0.94	1.00 (0.83 to 1.21)	0.98	1.61 (0.91 to 2.73)	0.090	1.87 (1.07 to 3.13)	0.022
Type-2 Diabetes Mellitus	1.00 (0.78 to 1.27)	0.98	0.94 (0.74 to 1.19)	0.61	0.77 (0.42 to 1.34)	0.37	0.92 (0.50 to 1.60)	0.77
Liver Risk Factors	1.29 (1.01 to 1.65)	0.041	1.25 (0.97 to 1.59)	0.076	0.50 (0.20 to 1.10)	0.11	0.62 (0.24 to 1.35)	0.27
Cirrhosis	1.40 (0.99 to 1.98)	0.054	1.33 (0.94 to 1.87)	0.10	2.90 (1.38 to 5.91)	0.004	3.64 (1.77 to 7.27)	<0.001
HIV	1.22 (0.94 to 1.56)	0.13	1.12 (0.87 to 1.44)	0.36	0.20 (0.01 to 1.03)	0.13	0.55 (0.03 to 2.67)	0.56
CCI >= 3	0.68 (0.56 to 0.83)	<0.001			12.3 (6.11 to 25.2)	<0.001		
FDep Q4 or Q5	0.94 (0.84 to 1.04)	0.21	0.93 (0.84 to 1.03)	0.17	1.21 (0.80 to 1.84)	0.36	1.30 (0.86 to 1.97)	0.21
Outbreak Period								
Pre-Outbreak [2013-2017[	—		—		—		—	
Outbreak [2017-June 2018[	1.52 (1.32 to 1.75)	<0.001	1.54 (1.34 to 1.77)	<0.001	0.85 (0.42 to 1.70)	0.66	0.74 (0.37 to 1.44)	0.38
Post-Outbreak [July 2018-2019]	1.46 (1.25 to 1.71)	<0.001	1.44 (1.23 to 1.69)	<0.001	0.96 (0.46 to 1.91)	0.90	1.08 (0.53 to 2.13)	0.83
COVID-19 [2020-June 2021]	1.21 (0.94 to 1.55)	0.13	1.17 (0.91 to 1.50)	0.21	1.36 (0.62 to 2.84)	0.43	1.82 (0.84 to 3.73)	0.11
Post-COVID-19 [July 2021-2024]	1.46 (1.26 to 1.70)	<0.001	1.44 (1.23 to 1.67)	<0.001	1.86 (1.07 to 3.30)	0.031	2.15 (1.25 to 3.77)	0.007

Abbreviations: CI = Confidence Interval, OR = Odds Ratio

B) Propensity-score matched models

Characteristic	Severe HAV (with CCI) OR (95% CI)	p-value	Severe HAV (w/o CCI) OR (95% CI)	p-value	HAV death (with CCI) OR (95% CI)	p-value	HAV death (w/o CCI) OR (95% CI)	p-value
Age per decade >30	1.00 (1.00 to 1.00)	0.31	1.00 (1.00 to 1.00)	0.62	1.00 (0.99 to 1.01)	0.75	1.00 (0.99 to 1.01)	0.86
Male sex	0.96 (0.86 to 1.07)	0.48	0.97 (0.87 to 1.09)	0.65	0.96 (0.62 to 1.50)	0.86	1.36 (0.89 to 2.11)	0.16
smoking	0.96 (0.80 to 1.14)	0.64	1.04 (0.87 to 1.24)	0.65	0.82 (0.41 to 1.52)	0.54	0.99 (0.51 to 1.81)	0.98
Type 2 diabetes mellitus	1.01 (0.80 to 1.27)	0.93	1.01 (0.80 to 1.27)	0.94	1.26 (0.68 to 2.22)	0.44	1.01 (0.57 to 1.70)	0.98
Liver risk factors	0.99 (0.78 to 1.24)	0.91	1.05 (0.83 to 1.32)	0.66	0.91 (0.37 to 1.91)	0.82	0.69 (0.27 to 1.53)	0.39
Alcohol use disorders	0.90 (0.72 to 1.12)	0.34	0.98 (0.78 to 1.21)	0.84	0.99 (0.48 to 1.91)	0.97	1.02 (0.52 to 1.92)	0.95
cirrhosis	1.18 (0.86 to 1.61)	0.29	1.08 (0.78 to 1.47)	0.65	1.24 (0.56 to 2.59)	0.58	0.86 (0.41 to 1.74)	0.69
obesity	0.94 (0.78 to 1.13)	0.53	0.95 (0.79 to 1.14)	0.60	0.89 (0.50 to 1.51)	0.67	0.98 (0.57 to 1.61)	0.93
HIV	1.01 (0.79 to 1.27)	0.96	0.90 (0.71 to 1.14)	0.39	0.70 (0.04 to 3.47)	0.73	0.94 (0.05 to 4.74)	0.95
CCI >= 3	0.81 (0.67 to 0.97)	0.021			0.85 (0.42 to 1.73)	0.65		
FDep Q4 or Q5	1.01 (0.91 to 1.12)	0.85	0.98 (0.88 to 1.08)	0.67	1.01 (0.67 to 1.52)	0.96	0.97 (0.65 to 1.46)	0.90
outbreak_period								
Pre-Outbreak [2013-2017[	—		—		—		—	
Outbreak [2017-June 2018[	1.42 (1.24 to 1.64)	<0.001	1.45 (1.26 to 1.66)	<0.001	0.78 (0.38 to 1.54)	0.48	1.03 (0.52 to 1.99)	0.93
Post-Outbreak [July 2018-2019]	1.49 (1.27 to 1.75)	<0.001	1.43 (1.22 to 1.68)	<0.001	0.98 (0.49 to 1.91)	0.96	0.84 (0.40 to 1.68)	0.63
COVID-19 [2020-June 2021]	1.28 (0.99 to 1.63)	0.054	1.14 (0.89 to 1.45)	0.30	1.03 (0.43 to 2.26)	0.94	1.54 (0.73 to 3.12)	0.24
Post-COVID-19 [July 2021-2024]	1.57 (1.35 to 1.83)	<0.001	1.41 (1.21 to 1.64)	<0.001	1.96 (1.14 to 3.45)	0.016	1.81 (1.07 to 3.12)	0.030

Abbreviations: CI = Confidence Interval, OR = Odds Ratio

Supplementary Figures

Figure S1: Study Flowchart

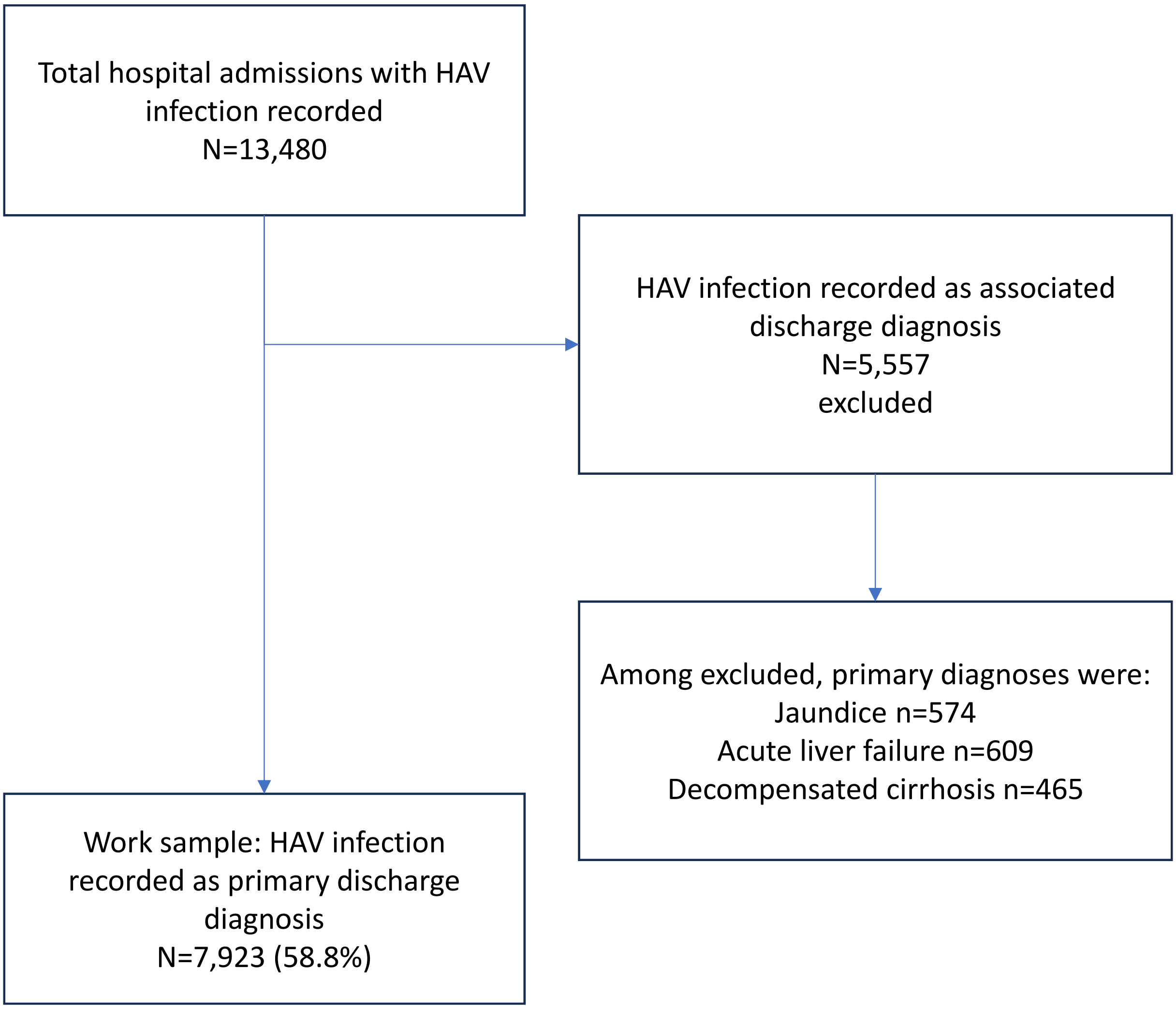

All patients with a primary or associated discharge diagnosis of hepatitis A (ICD-10 code B150 or B159) between January 2013 and December 2024 were considered. To increase specificity, only patients with Hepatitis A recorded as the primary discharge diagnosis (58.8%) were retained for analysis.

Figure S2: Quarterly number of HA cases recorded in hospitals (total, severe and deaths), France, 2013-2024, n=7,923 inpatients

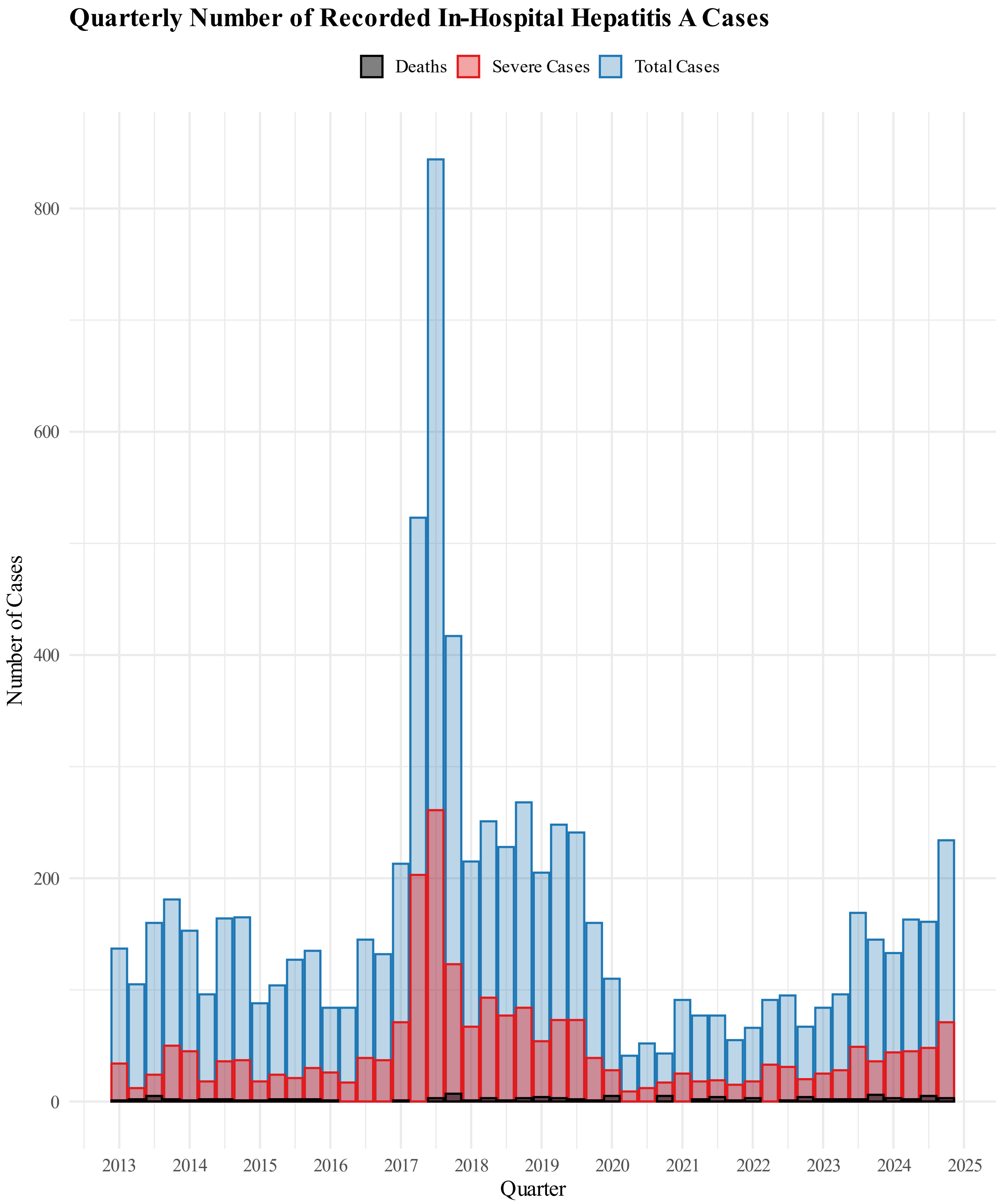

Total number of HA cases (blue), of severe cases (red) and of HA-related deaths (black) are shown. Two periods are highlighted: the outbreak among MSM (blue) and the COVID-19 pandemic (green).

Figure S3: Covariate balance after propensity score matching for severe Hepatitis A (A) and Hepatitis A-Related Death (B), French Hospitals, 2013-2024

A)

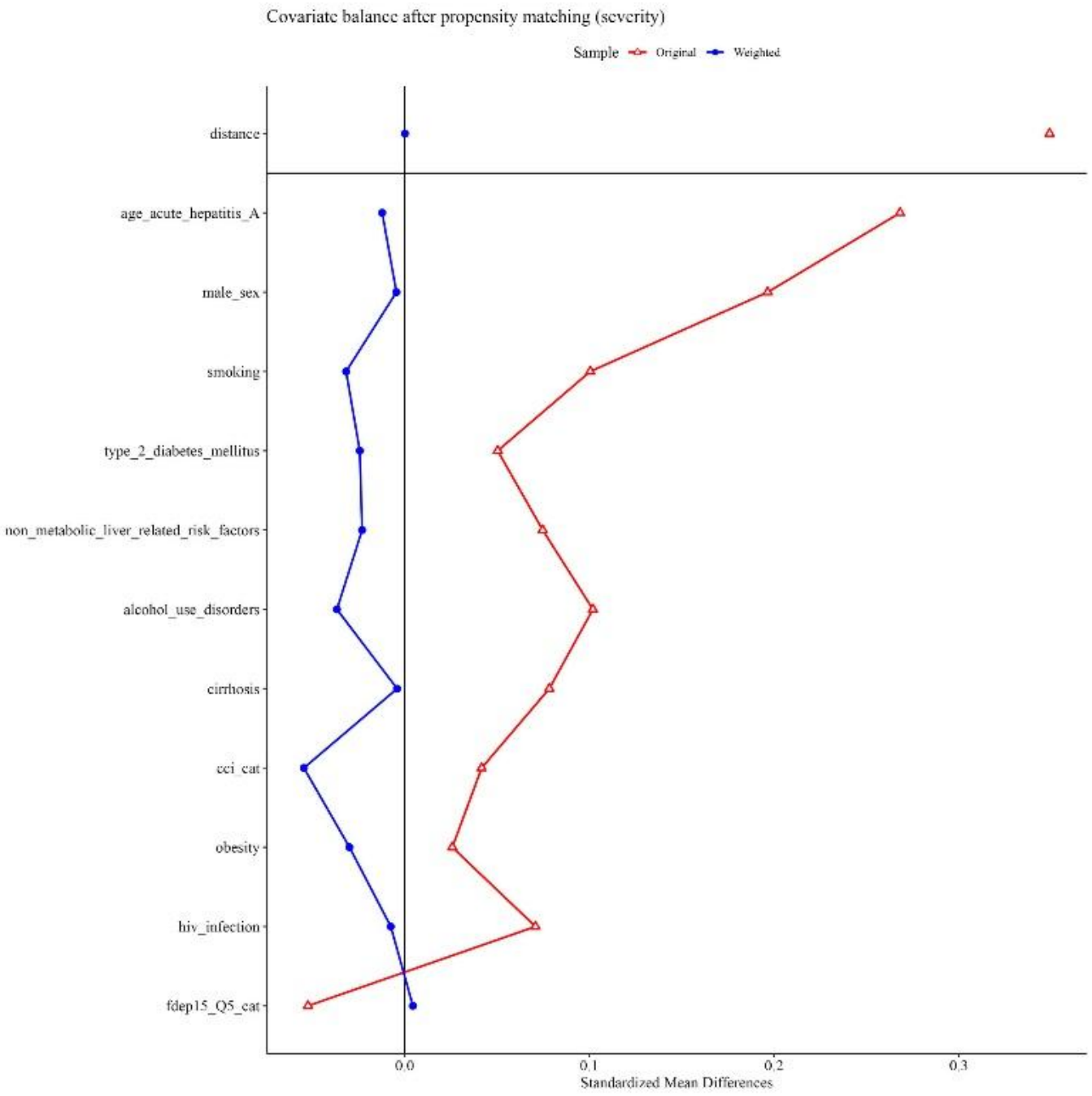

B)

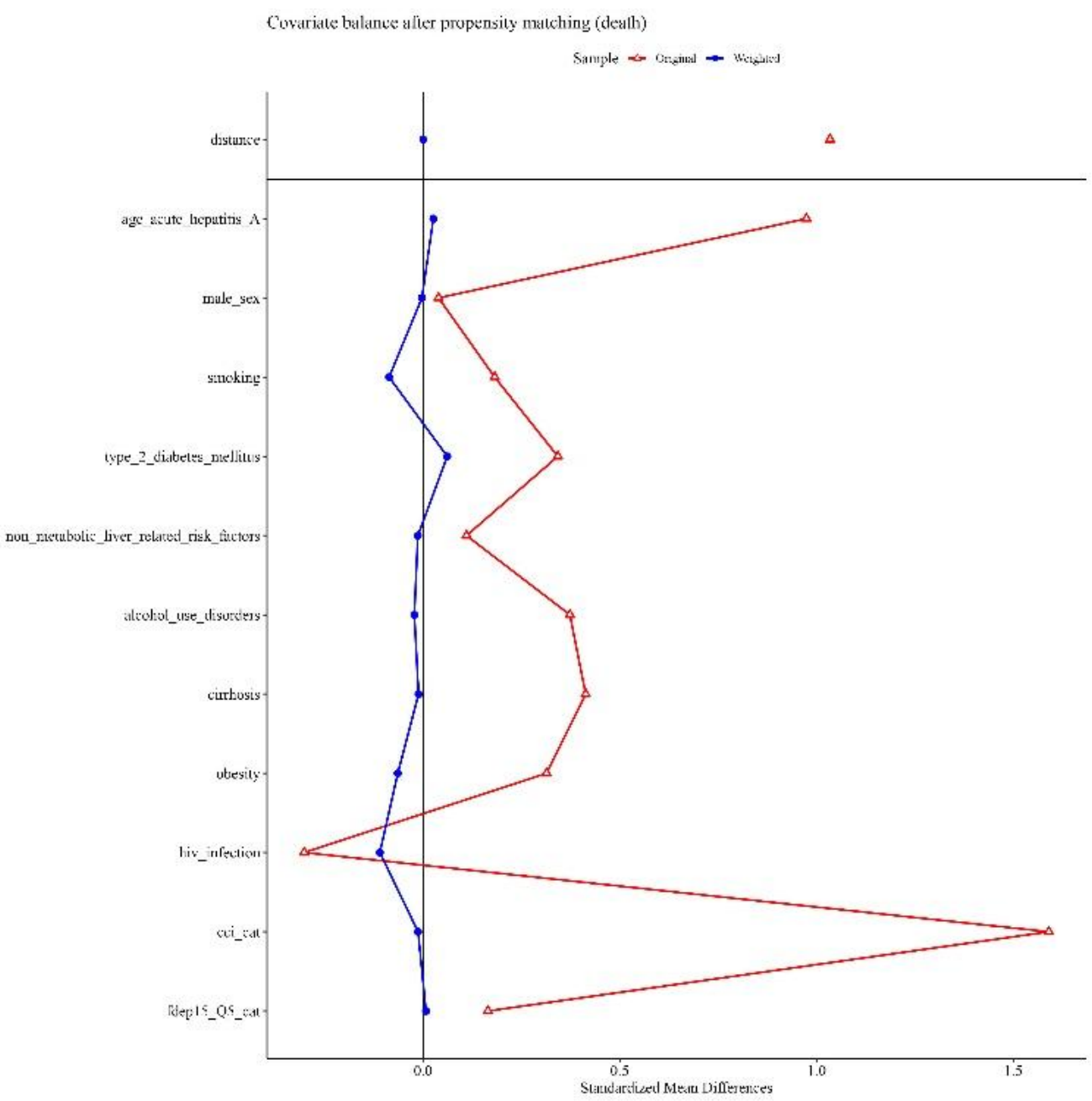

Supplement: Supplementary file 1 [file Supplementary_material_2500858.pdf]
